# Picture quiz

**Published:** 2023-12-01

**Spot the error(s), if any, in these images. Say what is wrong in each case (select as many options as needed).**

**1. Suture ready for forehand pass for right-handed surgeon**
*Select as many options as needed:*
  a. Everything is correct.
  b. The wrong part of the needle is being gripped
  c. The wrong part of the needle holder is used
  d. The needle faces the wrong direction
  e. The tip of the needle holder is curved/bent in the wrong direction, relative to the needle.
    **Figure:**
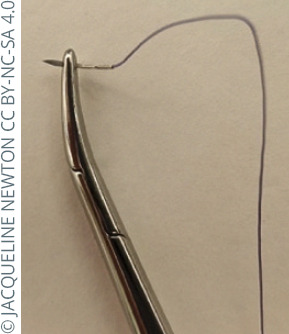
**2. Suture ready for forehand pass for left-handed surgeon**
*Select as many options as needed:*
  a. Everything is correct.
  b. The wrong part of the needle is being gripped
  c. The wrong part of the needle holder is used
  d. The needle faces the wrong direction
  e. The tip of the needle holder is curved/bent in the wrong direction, relative to the needle.
    **Figure:**
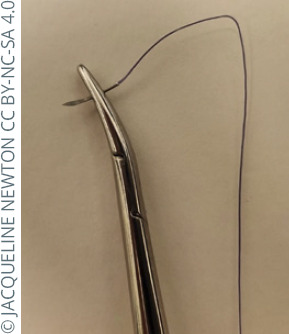
**3. Suture ready for forehand pass for right-handed surgeon**
*Select as many options as needed:*
  a. Everything is correct.
  b. The wrong part of the needle is being gripped
  c. The wrong part of the needle holder is used
  d. The needle faces the wrong direction
  e. The tip of the needle holder is curved/bent in the wrong direction, relative to the needle.
    **Figure:**
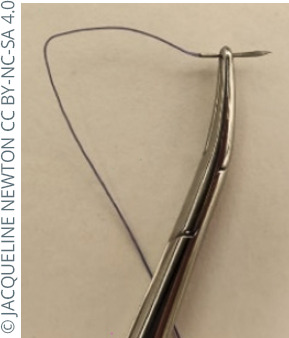
**4. Suture ready for forehand pass for left-handed surgeon**
*Select as many options as needed:*
  a. Everything is correct.
  b. The wrong part of the needle is being gripped
  c. The wrong part of the needle holder is used
  d. The needle faces the wrong direction
  e. The tip of the needle holder is curved/bent in the wrong direction, relative to the needle.
    **Figure:**
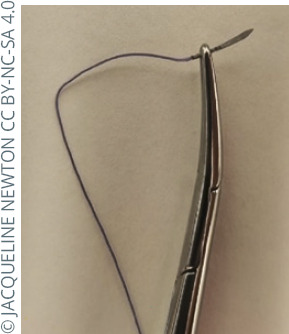

## ANSWERS

1 b. The needle is gripped too close to the point.
2. c, d, e. The tip of the needle holder should not curve/bend in the direction of the tip of the needle – it should curve/bend towards the swage.
3. d. The image is correct for a left-handed surgeon making a forehand pass. For a right-handed surgeon, the needle must point to the left, and the tip concave curve of the needle holder must curve to the right.
4. b. The needle is being gripped too close to the swage.

